# Molecular methods to diagnose pertussis: a case of confusion with *Bordetella bronchiseptica*

**DOI:** 10.1128/asmcr.00084-24

**Published:** 2025-09-09

**Authors:** Christopher M. Zarbock, Esse M. Evbuomwan, Melissa Shenep, Gautam Dantas, Rebekah E. Dumm

**Affiliations:** 1 Department of Pathology and Immunology, Division of Laboratory and Genomic Medicine, Washington University School of Medicine 12275, St. Louis, Missouri, USA; 2 The Edison Family Center for Genome Sciences and Systems Biology, Washington University School of Medicine 12275, St. Louis, Missouri, USA; 3 Department of Biomedical Engineering, Washington University in St. Louis 7548, St. Louis, Missouri, USA; 4 Department of Pediatric Infectious Diseases, Washington University School of Medicine 12275, St. Louis, Missouri, USA; Rush University Medical Center, Chicago, Illinois, USA

**Keywords:** *Bordetella*, *pertussis*, *bronchiseptica*, multiplex NAAT, respiratory pathogens

## Abstract

**Background:**

Prompt identification of clinically relevant *Bordetella* (e.g., *B. pertussis* and *B. parapertussis*) is critical for both treatment and isolation decisions to mitigate transmission among close contacts. Due to the organisms’ fastidious growth requirements, nucleic acid amplification tests (NAATs) have become a mainstay for identification and differentiation of *B. pertussis* and *B. parapertussis*. For NAATs detecting these closely related species, the nucleic acid target dictates assay sensitivity and specificity and varies between commercially available platforms.

**Case Summary:**

This case report describes a child’s course of cough and post-tussive emesis following bone marrow transplant for an underlying immunodeficiency. While an initial BioFire Respiratory Panel 2.1 (BioFire Diagnostics, LLC, Salt Lake City, UT, USA) detected *B. pertussis* DNA, a subsequent maxillary sinus culture grew *B. bronchiseptica*, identified by MALDI-TOF MS and whole-genome sequencing. Furthermore, on two subsequent occasions, nasopharyngeal specimens were positive for *B. pertussis* on the BioFire RP2.1; however, they were negative for *B. pertussis* on the DiaSorin Molecular Simplexa Bordetella Direct (DiaSorin Molecular LLC, Cypress, CA, USA). These seemingly disparate results can be explained by molecular target differences on the respective platforms and a rare instance of cross-reactivity with the genetic target for *B. pertussis* (*ptxP*) in an isolate of *B. bronchiseptica*.

**Conclusion:**

This case, where the presence of *ptxP* was confirmed by whole genome sequencing in an isolate of *B. bronchiseptica*, highlights a caveat to the molecular diagnosis of pertussis and emphasizes the importance of understanding gene targets to interpret results when false positive detections are suspected.

## INTRODUCTION

*Bordetella* is a genus of small, aerobic, gram-negative coccobacilli that can cause respiratory tract infections (1). Several clinically relevant species in this genus include *B. pertussis* and *B. parapertussis*, the causative agents of whooping cough, as well as *B. holmesii* and *B. bronchiseptica*. Of note, *B. pertussis* is highly infectious (1, 2), can cause severe disease in infants (2), and requires reporting to public health agencies. Isolation of *B. pertussis* requires culture with specialized media, so routine detection in clinical laboratories relies on nucleic acid amplification testing (NAAT). Molecular assays can be targeted to detect *B. pertussis* alone, to detect closely related organisms (e.g., *B. pertussis* and *B. parapertussis*), or can be multiplexed with other respiratory pathogens in a syndromic panel. Respiratory pathogen multiplex panels are convenient for the diagnosis of patients with respiratory illness of unclear etiology, given overlapping signs and symptoms. While such testing casts a wide net, it is important to recognize the limitations of multiplex assays and to interpret the results in the appropriate clinical context. This case report illustrates a potential pitfall of molecular methods to differentiate between closely related species of *Bordetella*, which was complicated by the use of a multiplex respiratory pathogen NAAT.

## CASE PRESENTATION

**Fig 1:**
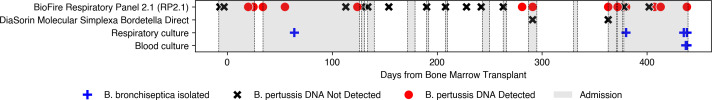
Timeline of the patient’s course including diagnostic detection of *Bordetella. B. pertussis* was detected by BioFire Respiratory Panel 2.1 (RP2.1) or the DiaSorin Molecular Simplexa Bordetella Direct, and *B. bronchiseptica* was isolated from respiratory or blood culture. Of note, each point represents a unique specimen (i.e., no repeat testing is reflected in these data).

In this case, a 3-year-old girl with underlying immunodeficiency underwent a 9/10 mismatched unrelated donor stem cell transplant. Nineteen days following her transplant, the patient developed a new-onset cough and had post-tussive emesis. The following day, a BioFire Respiratory Panel 2.1 (RP2.1) was performed on a nasopharyngeal (NP) swab (Fig. 1). This detected the nucleic acid of Rhinovirus/Enterovirus and *B. pertussis* for which the patient was given a 5-day course of azithromycin. The patient initially improved and was discharged on day 26 but was re-admitted on day 34 with worsening cough, post-tussive emesis, and fever. A repeat BioFire RP2.1 was performed and was positive for Adenovirus in addition to Rhinovirus/Enterovirus and *B. pertussis*. During this admission, the patient again tested positive for *B. pertussis* on the BioFire RP2.1 on day 55. As a part of a subsequent fever workup during this admission, CT scans of her sinuses were obtained on day 62, which were concerning for invasive fungal sinusitis. The patient underwent endoscopic sinus surgery to biopsy the area of concern and a sample was sent for culture. The Gram stain showed no polymorphonuclear leukocytes or organisms, and the culture ultimately grew few *B. bronchiseptica* on blood and chocolate agar*,* identified by MALDI-TOF mass spectrometry (RUO BDAL Revision H library, Bruker, MA, USA). She received 7 days of meropenem and 7 days of doxycycline to complete a 14-day course for sinusitis secondary to *B. bronchiseptica*. Several months later, on days 291 and 363, NP specimens tested positive for *B. pertussis* on the BioFire RP2.1, but negative for *B. pertussis* on a targeted *B. pertussis*/*B. parapertussis* NAAT, the DiaSorin Molecular Simplexa Bordetella Direct. She initially received a second 5-day course of azithromycin; however, due to stable respiratory symptoms with continued intermittent positivity of *B. pertussis* on the BioFire RP2.1, she did not receive a second treatment course given concerns for chronic colonization with *B. bronchiseptica* instead of *B. pertussis* infection. During a subsequent admission on days 437 and 438, *B. bronchiseptica* also grew out from multiple blood cultures. Ultimately, the patient expired due to multiple causes, including graft versus host disease and multi-system organ failure.

## DISCUSSION

*Bordetella* species can cause respiratory tract infections in both humans and animals (1). *B. pertussis* causes whooping cough and is a strict human pathogen with no known animal or environmental reservoir (3). *B. bronchiseptica* causes kennel cough in dogs and rarely human infections primarily in immunocompromised hosts. While *B. bronchiseptica* is typically considered an asymptomatic colonizing organism in humans, in the rare cases where it is thought to cause disease, it can be difficult to clear even with antibiotics (1). Molecular detection of *Bordetella* species by NAAT includes two common targets for *B. pertussis*: the pertussis toxin promoter (*ptxP*) and insertion sequence 481 (*IS481*) (Table 1). While *ptxP* provides high specificity for *B. pertussis* as it is only found in this organism (absent from the closely related *B. holmseii*, *B. bronchiseptica*, and *B. parapertussis*), assays utilizing this target demonstrate lower sensitivity for *B. pertussis* given that there is only a single copy of this target per isolate (2). This is the target utilized by the RP2.1 platform to detect *B. pertussis*. On the other hand, *IS481*—the target used by the Simplexa *Bordetella* Direct assay—provides increased sensitivity for *B. pertussis* as there are about 200 copies of this target per isolate (2). However, assays utilizing this target can demonstrate decreased specificity for *B. pertussis* as *IS481* is also found in *B. holmesii* and *B. bronchiseptica*.

**TABLE 1:** Comparison of molecular targets for *B. pertussis* between the BioFire Respiratory Panel 2.1 and the DiaSorin Molecular Simplexa *Bordetella* Direct^*a*^

	BioFire Respiratory Panel 2.1 (RP2.1)	DiaSorin Simplexa *Bordetella* Direct
*B*. *pertussis* target	*ptxP*	*IS481*
Copies per isolate		
*B*. *pertussis*	1	~200
*B*. *holmesii*	0	~10
*B*. *bronchiseptica*	0	<5 (<1% of isolates)
*B*. *parapertussis*	0	0 (4–6)
Overall sensitivity	Lower	Higher
Overall specificity	Higher	Lower

^*a*^ Portions of the table were adapted from reference 2 with permission.

**Fig 2:**
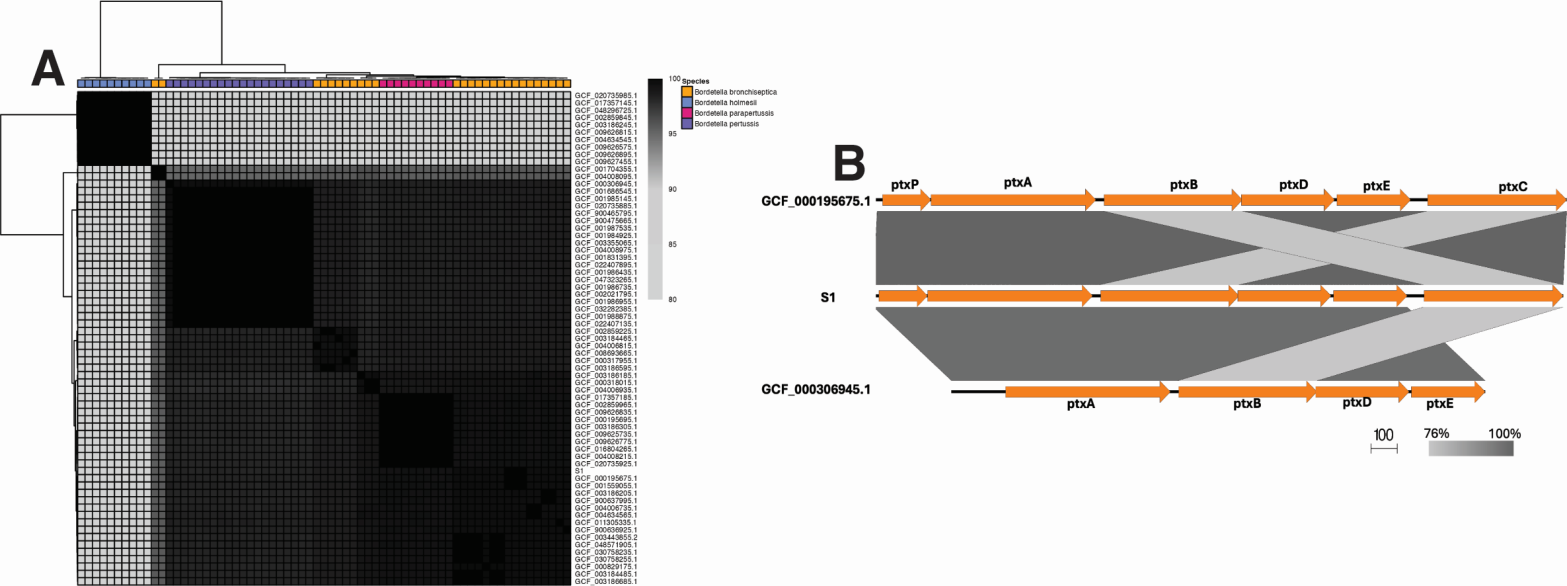
Comparative genomic analysis of patient isolate S1 validates *B. bronchiseptica* species identification and the presence of ptxP gene. (**A**) Pairwise whole-genome ANI of S1 compared to all 26 RefSeq deposited complete *B. bronchiseptica* genomes, 20 *B. pertussis* (GCF_000306945.1), 10 *B. parapertussis* (GCF_020735925.1), and 10 *B. holmesii* (GCF_020735985.1) complete genomes. RefSeq IDs in parentheses indicate type strains. (**B**) Comparison of the ptx locus from *B. bronchiseptica* RB50 (GCF_000195675.1), S1, and *B. pertussis* type strain 18323. The ptxP gene in S1 is 100% identical to the promoter region of RB50 and exhibits 94% nucleotide identity to *B. pertussis* type strain 18323.

In this case, *B. pertussis* DNA was detected on numerous occasions by the BioFire RP2.1, but both respiratory and blood cultures grew *B. bronchiseptica* as identified by MALDI-TOF MS. These results are seemingly disparate—especially since the RP2.1 target is thought to be highly specific for *B. pertussis*. However, the package insert for the BioFire RP2.1 indicates that the *ptxP* pertussis toxin pseudogene sequences can rarely be present in *B. bronchiseptica* and *B. parapertussis* (7). To confirm the presence of the *ptxP* gene within the *B. bronchiseptica* isolate isolated from the patient’s blood cultures (Fig. 2, isolate S1), genomic DNA was extracted using the QIAamp BiOstic Bacteremia DNA Kit (QIAGEN, Germantown, MD, USA). Whole-genome sequencing was performed with the Nextera XT DNA Library Preparation Kit (Illumina, San Diego, CA, USA) on the NovaSeq X platform (Illumina). *De novo* genome assembly was constructed using Unicycler (8) v0.5.0 Illumina-only assembly process and then annotated using Bakta (9) v1.9.4. Pairwise whole-genome average nucleotide identity (ANI) was calculated between the assembled genome of S1 and publicly available *Bordetella* genomes using FastANI (10) v1.33 and plotted using pheatmap (11) v1.0.12 in R v4.4.2. S1 exhibited an ANI greater than 95% with all *B. bronchiseptica* genomes available in the RefSeq database (Fig. 2A), confirming its species identity (12). Next, using EasyFig (13) v2.2.5, the pertussis toxin gene locus of this clinical isolate (S1) was compared with *B. pertussis* strain 18323 and the annotated genome of *B. bronchiseptica* RB50. In this analysis of S1, the pertussis toxin promoter (*ptxP*) sequence was identified upstream of pertussis toxin subunit 1 (*ptxA*) (Fig. 2B). A nucleotide blast revealed that this sequence shared 100% identity with the *ptxP* sequence of *B. bronchiseptica strain* ATCC 786 (14) (GenBank: AF157362.1), 100% identity with the promoter region upstream of the *ptxA* gene in *B. bronchiseptica* RB50 (15) and 94% identity with the *ptxP* sequence of *B. pertussis* type strain 18323 (GenBank: HE965805.1; RefSeq: GCF_000306945.1 [16]) and *B. pertussis* ATCC 9340 (14) (GenBank: AF157343.1). *IS481* was not identified using ISfinder (17). In order to further validate this finding, we aligned reads from S1 to three reference sequences containing IS481 using Bowtie2 (18) (accession: KF871114.1, M28220.1, and AB670737.1). None of the reads mapped to M28220.1, which only contained the IS481 sequence, further supporting that IS481 is absent in this isolate. Furthermore, only 0.03% of the reads mapped to the other references.

Given the culture growth of *B. bronchiseptica* identified by MALDI-TOF, and the presence of pertussis toxin promoter (*ptxP*) sequence in the clinical isolate confirmed by sequencing to be *B. bronchiseptica*, we present this as an example of false positive detection of *B. pertussis* on the BioFire RP2.1 assay. Furthermore, in two instances where *B. pertussis* DNA was detected by the BioFire RP2.1 assay, it was not detected on the concurrent Simplexa Bordetella Direct assay, which uses *IS481* as its *B. pertussis* target (19).

In this case, the multiplexed BioFire RP2.1 was appropriately utilized in the diagnosis of a respiratory illness in an immunosuppressed individual given its breadth of pathogen coverage. The BioFire RP2.1 limitations most notably include decreased sensitivity due to the use of *ptxP* as a target, and, as such, the package insert recommends the use of a more sensitive targeted assay in cases where pertussis is suspected (7). However, when the care team received the result that *B. pertussis* DNA was detected, it was consistent with the clinical picture given the post-tussive emesis, which is often associated with whooping cough (20). In this case, *B. bronchiseptica* eventually grew in the maxillary sinus respiratory culture, which prompted the clinical team to change their antibiotic management; however, this was approximately 1 month following the initial *B. pertussis* positive BioFire RP2.1 assay.

In summary, following the growth of *B. bronchiseptica* in bacterial culture, the possibility of cross-reactivity was discussed with the care team. The presence of a *ptxP* pseudogene and the likely absence of *IS481* in this case’s isolate of *B. bronchiseptica* explain the numerous BioFire RP2.1 assay false positives for *B*. pertussis DNA and the two concurrent Simplexa Bordetella Direct true negatives for *B. pertussis* DNA. This case highlights a rare case of cross-reactivity of *B. bronchiseptica* with *B. pertussis* targets on the BioFire RP2.1. No single target for *B. pertussis* demonstrates ideal sensitivity and specificity; thus, this case underscores the importance of understanding the gene targets utilized to aid in interpretations when discrepant detections are suspected.

## Data Availability

Raw sequence data and genomic assembly generated from this paper are available at BioProject PRJNA1268506.
